# The impact of collaborative atmosphere on innovative work behavior of college teachers, North China

**DOI:** 10.3389/fpsyg.2024.1497503

**Published:** 2025-01-14

**Authors:** Qian Liu, Yu Sun

**Affiliations:** ^1^School of Marxism, Hengshui University, Hengshui, Hebei, China; ^2^Chinese International College, Dhurakij Pundit University, Bangkok, Thailand

**Keywords:** collaborative climate, educators’ innovative work behavior, knowledge sharing, knowledge management, Chinese teacher

## Abstract

Innovation and progress serve as the driving forces behind national development. Universities, with their comprehensive academic systems and robust research capabilities, undoubtedly play a crucial role in fostering student innovation and advancing faculty research innovation. This study aims to explore the relationship between the collaborative climate and innovative work behavior of university educators, as well as the mediating effect of knowledge sharing, in order to provide an important theoretical basis for universities to better promote innovative work behavior. This study adopts questionnaire survey method and semi-structured interview method. In the questionnaire survey stage, this study uses 473 in-service educators in colleges and universities as the research objects; in the interview stage, this study uses 8 in-service educators as the research objects. The results of the questionnaire study prove that educators’ cooperation atmosphere has a significant positive impact on innovative work behavior, educators’ collaborative climate has a significant positive impact on knowledge sharing, knowledge sharing has a significant positive impact on innovative work behavior, and knowledge sharing has a significant positive impact on teachers’ collaborative climate. There is a significant mediating effect on employees’ innovative work behavior. The interview results found that a positive collaborative climate within universities can influence teachers’ innovative work behavior through three channels: colleague support, management resource provision, and academic freedom encouragement. Therefore, a positive collaborative climate not only encourages communication and cooperation among faculty members but also inspires them to adopt and develop new methods and technologies in their research and teaching practices. Universities should place greater emphasis on enhancing their internal collaborative atmosphere.

## 1 Introduction

Innovation is one of the key topics of global interest in the era of the knowledge economy. It serves not only as a crucial source of core competitiveness for organizations, regions and countries, but also as a significant driving force for the progress of human society (Delgado et al., 2020). Nowadays, nations worldwide have been accelerating their innovational efforts, striving for further achievements in the next ears of technological revolutions as well as industrial transformations. Innovation has become the centerpiece of this global tech competition (Pian et al., 2019). Universities and colleges are essential in nurturing innovative individuals, and the innovative behaviors of educators within these institutions are directly influential to important aspects such as the standard of higher education, the quality of research outputs, and development of their students (Tassabehji et al., 2019).

Innovative advancements are sources for domestic development, and universities, with their comprehensive disciplinary systems and profound research capabilities, undoubtedly play a crucial role in cultivating student innovation and academic research innovation (Chedid et al., 2020). Despite their sustainable operations, universities still face pressures to evolve, modernize, and alter their practices and services to meet the various requirements from societal needs, competitive demands, and new technologies (Hall and Lulich, 2021). Since the beginning of this century, research on innovation has gradually shifted its focus from organizational levels to microscopic level, with increasing attention on individual innovative work behaviors (Rese et al., 2020). University educators, distinct from corporate researchers, not only shoulder the burden of research, but also undertake responsibilities from teaching and innovative practices within academic research (Arsawan et al., 2022).

Innovation serves as the first driving force for leading positive progress of universities and colleges. In order to advance further, these institutions have to place innovation at the core of their strategic development, while educators serve as pivotal parts realizing these innovations. On one hand, university educators need to continually integrate resources and absorb new knowledge in response to rapidly updating technological information, innovating in both research content and methodologies to maintain their institution’s competitiveness in research fields (Almulhim, 2020); on the other hand, educators should also adopt new educational principles during teaching, update their course contents, and apply innovative teaching methods and tools. This approach not only provides students with newest educational materials but also diverse strategies for analyzing and solving problems (Crupi et al., 2021). Therefore, the innovative work behavior of educators refers to the process of generating, introducing, and implementing beneficial and novel ideas in both research and teaching, incorporating both the formation and execution of these ideas (Zeb et al., 2020). Educators with innovative work behaviors are able to work creatively, delivering positive outcomes for their organizations (Izzati, 2018). Studying the mechanisms influencing educators’ innovative work behaviors is crucial for the development of individuals in universities, the quality of scientific research and teaching, and the implementation of innovation-driven development strategies (Li et al., 2019).

Harrison and Brodeth (1999) regard the construction of a collaborative climate as a key principle for maintaining the development of learning and research in higher education institutions. According to Tillman (2006), the collaborative climate in higher education is largely an unwritten yet deeply rooted collaborative process involving research, teaching, guidance, and service; this process manifests at various levels within universities, including individuals, departments, schools, and disciplines. Furthermore, research by Diamantes et al. (2002) indicates that effective collaborative work with colleagues is crucial for faculty members aiming for promotions and tenure. Therefore, this study considers exploring the impact of collaborative climate on Educators’ innovative work behavior.

The purpose of knowledge management is to provide the right information to the right people at the right time (Snowden, 2000). According to knowledge management theory, success within schools depends on knowledge sharing among educators and the resulting behavioral outcomes (Hislop, 2013). Based on this theory, organizational knowledge creation relies on the internal reintegration of knowledge (Lannon and Walsh, 2019). This approach to knowledge management can systematically influence knowledge exchange, application, and creation, thus generating value (Kozhakhmet and Nazri, 2017; Li et al., 2009). Strategies for knowledge management in higher education institutions can lead to positive subsequent behaviors stemming from knowledge sharing, as collaboration is the foundation of innovation, and knowledge sharing is at the focal point between collaborative climate and educators’ innovative work behavior (Chen et al., 2009). Therefore, this study, grounded in knowledge management theory, seeks to explore the mediating role of knowledge sharing in the relationship between collaborative climate and educators’ innovative work behavior.

In the field of research on educators’ innovative work behaviors, past studies have predominantly adopted a singular quantitative approach, as seen in the works of Johari et al. (2021) and Karavasilis (2019), which implemented survey methodologies. However, Hosseini and Haghighi Shirazi (2021) have pointed out that relying solely on surveys as a data collection tool may have limitations. Solely quantitative data might not fully represent the genuine opinion of participants, as their responses could be affected by various biases, leading to less authentic responses. To overcome this limitation, Hosseini and Haghighi Shirazi (2021) recommend that researchers adopt a mixed-methods design, combining quantitative and qualitative approaches to provide a more comprehensive, in-depth, and authentic description of the questions. Therefore, this study employs a mixed-methods approach to more accurately understand the complexity and diversity of educators’ innovative work behaviors.

This study has two main contributions. First, it applies the theoretical framework of knowledge management to the field of education, which helps to test and develop the applicability of knowledge management theory in educational contexts. Knowledge management aims to continuously acquire and update knowledge to achieve organizational knowledge creation (Claver-Cortés et al., 2018; Kaschig et al., 2016). Understanding how knowledge sharing mediates the relationship between collaborative climate and educators’ innovative work behavior will enhance the understanding of the processes of knowledge flow and transformation within organizations, contributing to a more detailed and in-depth exploration of the mechanisms of knowledge flow and transformation in knowledge management theory. Second, by reviewing the related research on collaborative climate and educators’ innovative work behavior, it is found that collaborative climate is considered an important contextual factor by scholars (Le et al., 2020). Knowledge sharing, as a mediating variable, starts from the perspective of individual educators, positing that sharing knowledge among individuals helps to enhance the organization’s knowledge reserves and fosters the exchange of ideas among educators, which directly or indirectly influences their level of innovation. This has theoretical significance for promoting innovation strategies in higher education institutions and faculty development. Moreover, the stricter requirements for innovative work behavior in higher education institutions make this research more representative, expanding the study of innovative work behavior.

Therefore, the main research objectives of this study are as follows:

1. To explore the impact of collaborative climate on educators’ innovative work behavior in higher education institutions in North China.
2. To investigate the influence of collaborative climate on knowledge sharing among educators in higher education institutions in North China.
3. To examine the effect of knowledge sharing on educators’ innovative work behavior in higher education institutions in North China.
4. To explore the mediating effect of knowledge sharing between collaborative climate and educators’ innovative work behavior in higher education institutions in North China.

## 2 Literature review

### 2.1 Theoretical foundation—knowledge management theory

Knowledge management focuses on the impacts of knowledge processes such as knowledge acquisition, accumulation, sharing, transformation, diffusion, and innovation (Andersen, 1999). Knowledge management encourages cooperation (Feiz et al., 2019) and emphasizes the creation of new knowledge through effective and efficient management of the knowledge within universities. Based on such theory, knowledge acquisition emphasizes the establishment of an environment which behaves conducive to educators’ access to knowledge, as well as their integration and re-creation (Cui et al., 2020). Universities can foster an active collaborative climate that facilitates both the external acquisition of knowledge and internal accumulation of resources among educator, enhancing the reciprocating and supportive relationships between them. Moreover, knowledge sharing, specifically referring to universities coordinating educators across disciplines to share their own knowledge and experience, where educators contribute and acquire knowledge simultaneously in this process (Van Den Hooff and De Ridder, 2004). Furthermore, knowledge innovation, based on the extensive exchange of educators’ knowledge, promotes innovation in knowledge and implements it as innovative work behavior (Zhao, 2010). Knowledge is introduced from its collaborative climate, and proceeds to spread to other members within the organization through the process of knowledge sharing. A positive collaborative climate encourages the sharing of knowledge, which drives innovative work behavior to a further extent. This innovative work behavior, in turn, brings new knowledge and experiences to the organization, creating a virtuous cycle. This model reflects the core idea of knowledge management is a continuous process and the acquisition, sharing, diffusion, and innovation of knowledge are interconnected.

### 2.2 The relationship between collaborative climate and innovative work behavior

Guided by a collaborative climate among university educators, collaboration naturally originates from educators themselves within a context of active interdependence. Educators not only develop shared values and common interests, as well as a united vision, but are also capable of accommodate diverse educational perspective, fostering their own development (Bhatti et al., 2021). For higher education institutions, collaboration climate among educators can profoundly influence its individuals through its strong nurturing capacity. Utilizing the collaborative platforms established by universities and colleges, innovative capabilities of educators are greatly enhanced by continual communications among them (Setini et al., 2020), resulting in sustained enthusiasm and actions toward further innovation. Hence, the theoretical hypothesis is proposed:

H1: The collaborative climate among educators in Chinese universities has a significant positive impact on their innovative work behavior.

### 2.3 The relationship between collaborative climate and knowledge sharing

A collaborative climate is considered as a fundamental prerequisite for promoting knowledge sharing among members within organizations (Hong et al., 2019). The positive impact of a collaborative organizational climate on knowledge sharing is of great significance; cultivating a cooperative environment within work teams that facilitates knowledge sharing among individuals is crucial (Wannapiroon and Pimdee, 2022). Collaborations between university faculties are based on resource sharing under the same purpose. The knowledge backgrounds, approaches of thinking, value orientations and academic expertise of different educators vary, thus, collaboration among them is beneficial for reciprocative learning, wisdom sharing, problem solving as well as the achievement of goals, eventually leading to value enhancement (Franco and Pinho, 2019). Therefore, the following theoretical hypothesis is proposed.

H2: The collaborative atmosphere among educators in Chinese universities has a significant positive impact on knowledge sharing.

### 2.4 The relationship between knowledge sharing and innovative work behavior

Knowledge sharing aims to enhance the utility value of educators’ knowledge resources, especially crucial for whether different subjects are able to conduct the transmission, transformation and innovation of knowledge, in order to provide guidance toward practical issues (Jian, 2019). The community of colleagues possesses rich resources necessary for educators’ development (García-Sánchez et al., 2019). The outcomes of knowledge sharing among educators include professional knowledge increase, personal value amplification and constant innovations (Hero and Lindfors, 2019). Educators interact with each other through activities such as lectures and researches, mentor-mentee pairing, collaborative lecture planning, and project research. Knowledge is shared and transferred during these educational events, and the divergent dialogues in groups represents interactions from different viewpoints and perspectives. New concepts are formed through educators’ critical thinking and reflections, eventually achieving educators’ individual innovation (Sharma and Sharma, 2021). Therefore, in universities, knowledge sharing is regarded as means for promoting innovative work behaviors among educators. Therefore the following theoretical hypothesis is proposed:

H3: Knowledge sharing among educators in Chinese universities has a significant positive impact on their innovative work behavior.

### 2.5 The relationship among collaborative climate, knowledge sharing, and innovative work behavior

According to knowledge management theory, through the reconstruction of knowledge systems within an organization, the entire process of optimizing knowledge collection, acquisition, sharing, applications, feedback, innovation and refinement is designed to continuously innovate knowledge within the organization, accumulate and enhance individual knowledge, and eventually convert all into intellectual capitals, which is manifested in innovative work behaviors, thereby promoting sustainable development (Kim, 2024). Specific implementation of knowledge acquisitions in universities may involve creating an atmosphere conducive to educators’ access to knowledge (Balle et al., 2020), and enhancing mutually supportive relationships among them; knowledge sharing specifically refers to the university coordinating educators across disciplines to share their knowledge and experiences, through which educators contribute to and acquire knowledge; knowledge innovation is based on the extensive knowledge communications among educators, promoting the recreation of knowledge and applying new knowledge output behaviors or results (Zhao, 2010). Collaboration of educators is based on the diverse wisdom each possesses, providing a foundation for their knowledge sharing, and in cooperative exchanges such as seminars, research meetings and other forms of cooperation, complementing strength to produce the best outputs, achieving the transmission and reconstruction of knowledge among subjects (Pellegrini et al., 2020).

Furthermore, Kucharska and Kowalczyk (2016) indicated that knowledge sharing serves as a bridge between collaborative climate and innovation. Organizations can focus on developing a collaborative climate to create an appropriate environment that encourages knowledge-sharing activities and interactions among individuals. This, in turn, motivates individuals to share more ideas, practices, and willingness, leading to the systematic transfer of knowledge and greater application of knowledge to innovative work behavior. Yang et al. (2018) found that knowledge sharing mediates the relationship between collaborative climate and innovation capability. The research by Chen et al. (2009) demonstrated that collaboration is the foundation of innovation in higher education institutions, with knowledge sharing being the central focus between collaboration and innovation. Therefore, the following theoretical hypothesis is proposed:

H4: Knowledge sharing in Chinese universities has a significant mediating effect between educators’ collaborative climate and innovative work behavior. Collaborative climate of educators has a significant mediating effect.

## 3 Research methods and design

### 3.1 Research framework

### 3.2 Research design

This study employs an approach of mixed methods that combines questionnaire surveys along with interview surveys. Although there are differences between quantitative and qualitative research, there is no conflict in between, whereas their integration helps to expand the inclusivity of the study, delve further depth into the research, and even offers the potential for the methods to complement each other. This research uses an explanatory design with mixed methods, which follows a sequence of quantitative processes followed by qualitative phases. Participants for subsequent phases are selected based on tracking and translating the results of the first phase, with qualitative outcomes aiding to interpret the quantitative findings. The interviews in this study are conducted with a semi-structured format, which is a common and effective method in qualitative research (Clarke and Braun, 2013).

### 3.3 Research subjects

The scope of this study focuses on the in-service teachers at a Double First-Class University and three regular comprehensive undergraduate institutions in North China. These four universities have achieved significant research and innovation results in Hebei Province and possess strong management systems, making them the “forerunners” in accelerating innovation in the region. In the first phase of this study, convenience sampling was used to distribute questionnaires to 500 university educators, yielding 437 valid responses. The 63 questionnaires that were discarded were due to the respondents’ answers being logically inconsistent, such as providing contradictory answers to consecutive questions. These questionnaires are considered invalid in this study. The effective response rate for this study’s questionnaire is 87.400%.

**Figure 1:**
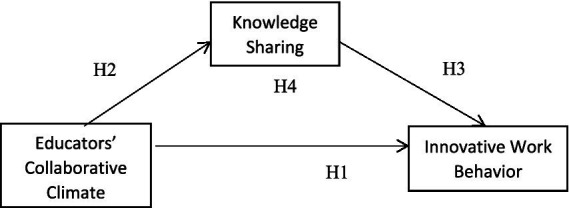
Research framework.

In the second phase, purposive sampling was used to select eight university educators with experience in innovative work as interview subjects to acquire their views and opinions on the relationship among educators’ collaboration climate, innovative work behavior, and knowledge sharing. The eight educators participating in the interviews were required to have at least 5 years of teaching or research experience. The study consulted the university’s administrative department and the teaching office to obtain a list of eligible educators and to make contact with them (Figure 1).

### 3.4 Research instruments

1. The scale for measuring the educator collaborative climate in universities was adopted from Tang (2017) and utilizes a Likert 5-point scoring method, where 1–5 correspond to “very rarely,” “rarely,” “uncertain,” “sufficiently,” and “excessively,” respectively. This scale consists of 14 items. In this study, the Cronbach’s Alpha for the educators’ collaborative climate scale is 0.958.
2. The scale for measuring innovative work behavior was designed by Hidayat and Patras (2022) and consists of a single dimension with 11 items. This scale also uses a Likert 5-point scoring method. In this study, the Cronbach’s Alpha for the innovative work behavior scale is 0.939.
3. The scale for measuring knowledge sharing was developed by Van den Hooff and De Ridder (2004) and consists of 10 items across two dimensions: knowledge contribution and knowledge acquisition. This study adopts a Likert 5-point scoring method and the Cronbach’s Alpha of knowledge sharing scale in this study is 0.898.
4. The interview outline for this study was originally drafted and then revised by five professionals with doctoral degrees in education and at least 5 years of teaching experience in higher education institutions before its formal implementations. See Table 1.

**Table 1:** Expert information form.

Expert	School	Title	Degree	Years of work	Field of specialization
T1	School A	Professor	PhD	15 Years	Educational Methodology
T2	School A	Associate Professor	PhD	7 Years	Modern Educational Technology and Educational Informatization
T3	School B	Professor	PhD	12 Years	Educational Policy Research
T4	School A	Assistant Professor	PhD	6 Years	Educational Philosophy
T5	School C	Associate Professor	PhD	10 Years	Educational Psychology

Questions are as follows:

1. What do you think about the collaborative climate at your university? From which aspects are collaborations evident? In terms of teaching and research, who provides you with help and support and how exactly?
2. What innovative ideas or behaviors have you had in your recent research and teaching? How were they inspired?
3. Do others at your university help and support you in generating innovative work behavior?
4. How do you share knowledge at your university on a daily basis? How do you adopt knowledge from others? What kind of assistance does your university provide in these processes of knowledge sharing and gaining?
5. Has knowledge sharing among colleagues brought you any innovative ideas or behaviors in your work? Or has the knowledge you shared inspired other colleagues?
6. Have you or your colleagues ever gained or contributed knowledge in a cooperative setting provided by the university that led to innovative work behaviors?

### 3.5 Research ethics

This specific section adheres to Article 20 and 21 of Section 3.2.2 in the “National Policy and Guidelines for Human Research” of the Thai National Research Council NROO, 2016 edition. During the research process, researchers must take measures to protect the personal and sensitive information of participants. It is necessary to ensure that participants are aware of the measures taken to protect their private information and records, and under what circumstances and by whom their private information and records will be accessed. This study has received an ethical review certificate from Dhurakij Pundit University (Certificate number: DPU_BSH 090166/2565).

## 4 Research results

### 4.1 Validity test

This study utilized confirmatory factor analysis to measure the convergent validity between different variables. After testing, all dimensions were found to exceed the standard values. See Table 2.

**Table 2:** Convergent validity test for variables.

Variable	Measurement entries	Factor load	CR	AVE
Educators’ collaborative climate	A1	0.727	0.945	0.553
	A2	0.737		
	A3	0.723		
	A4	0.755		
	A5	0.759		
	A6	0.752		
	A7	0.745		
	A8	0.756		
	A9	0.749		
	A10	0.724		
	A11	0.748		
	A12	0.736		
	A13	0.762		
	A14	0.732		
Innovative work behavior	B1	0.731	0.910	0.502
	B2	0.660		
	B3	0.707		
	B4	0.737		
	B5	0.686		
	B6	0.713		
	B7	0.694		
	B8	0.695		
	B9	0.746		
	B10	0.712		
Knowledge contribution	C1_1	0.746	0.854	0.540
	C1_2	0.690		
	C1_3	0.724		
	C1_4	0.761		
	C1_5	0.753		
Knowledge acquisition	C2_1	0.735	0.851	0.534
	C2_2	0.777		
	C2_3	0.745		
	C2_4	0.714		
	C2_5	0.680		

In the discriminant validity test, it is considered valid if the square root of the Average Variance Extracted (AVE) is greater than the correlation coefficients between any pair of variables. Thus, this study confirms that there is discriminant validity among the variables, as detailed in Table 3.

**Table 3:** Discriminant validity analysis.

	M	SD	Educators’ collaborative climate	Innovative work behavior	Knowledge contribution	Knowledge acquisition
Educators’ collaborative climate	3.338	0.842	0.743			
Innovative work behavior	3.201	0.767	0.655***	0.708		
Knowledge contribution	3.308	0.827	0.499***	0.464***	0.735	
Knowledge acquisition	3.280	0.833	0.491***	0.416***	0.440***	0.731

^***^p < 0.001.

Diagonal values represent the AVE values of the constructs; values below the diagonal show the correlation between variables.

### 4.2 Correlation analysis

There is a positive correlation between educators’ collaborative climate, innovative work behavior, knowledge contribution, knowledge acquisition and knowledge sharing. Results of correlation analysis are specifically shown in Table 3.

### 4.3 Structural equation testing

This section obtains the fit indices for structural equation model constructed in the study. The CMIN value is 394.132, and the CMIN/DF value is 1.332, which is less than 3, indicating a good fit for the structural equation model in this study. The RMSEA value is 0.028, which is below the standard value (0.100). The NFI value is 0.940, the CFI value is 0.984, and the IFI value is 0.984. All these values are within standard ranges, which also demonstrates that the structural equation model fits well and that further analysis of direct and indirect effects can be performed (Table 4).

**Table 4:** Fit indices for confirmatory factor analysis model.

Common indices	CMIN/DF	RMSEA	CFI	NFI	IFI
Judgment criteria	<3	<0.100	>0.900	>0.900	>0.900
Value	1.332	0.028	0.984	0.940	0.984

Data source: sorted and compiled by the current study.

Based on the SEM Path Analysis in Table 5, educator collaborative climate has a significant positive effect on innovative work behavior (*B* = 0.398, *β* = 0.420, *p* < 0.001); educator collaborative climate has a significant positive effect on knowledge sharing (*B* = 0.536, *β* = 0.766, *p* < 0.001); knowledge sharing has a significant positive effect on innovative work behavior (*B* = 0.507, *β* = 0.373, *p* < 0.001).

**Table 5:** SEM path analysis.

Path	Unstandardized path coefficient	S.E.	C.R.	*p*	Standardized path coefficient
Innovative work behavior ← educator collaborative climate	0.398	0.091	4.380	0.000	0.420
Knowledge sharing ← educator collaborative climate	0.536	0.049	10.862	0.000	0.766
Innovative work behavior ← knowledge sharing	0.507	0.149	3.391	0.000	0.373

Date Source: sorted and compiled by the current study.

### 4.4 Bootstrap mediation effect testing

In this study, the Bootstrap sampling method was utilized to test the mediation effects, with the number of samples set to 5,000 (Hesterberg, 2015). The results show that the mediation effect of educator collaborative climate → knowledge sharing → innovative work behavior is significant (standardized coefficient = 0.286, *p* = 0.002, and the 95% confidence interval between 0.123 and 0.555 does not include zero), and the model’s direct effect is significant (standardized coefficient = 0.420, *p* < 0.001, and the 95% confidence interval between 0.148 and 0.620 does not include zero). The total effect of the model is also significant (standardized coefficient = 0.705, *p* < 0.001, and the 95% confidence interval between 0.628 and 0.773 does not include zero), indicating that this mediation model represents partial mediation.

### 4.5 Interview analysis results

Since the purpose of the interview study is to further explain and deepen the quantitative research results, the interview questions were constructed based on the theoretical framework of the study (in this research, knowledge management theory) to establish preliminary definitions and variable relationships, and the interview questions were designed around this framework (Fereday and Muir-Cochrane, 2006). This interview utilized a semi-structured format, which is a common and effective method in qualitative research (Clarke and Braun, 2013). In this format, the study prepared an interview outline in advance to guide the direction of the conversation and ensure that the interview could focus on the core topics of the research. However, the study did not strictly limit the wording of the questions or the order in which they were asked, as qualitative interviews emphasize in-depth communication and natural dialogue. The study employed thematic analysis to analyze the interview content and used a three-level coding system to deeply explore the information within the data.

#### 4.5.1 Initial concepts

During the process of open coding, each interviewee’s responses were read carefully and specifically. The purpose of this stage was to identify key ideas, viewpoints and themes of the interviews. By carefully analyzing the content of the interviews, specific description and viewpoints regarding the collaborative climate, innovative behaviors, and knowledge sharing among educators were highlighted. Each marked piece of content was regarded as an independent opinion. In order to organize and interpret these data in a better way, these opinions were categorized and labeled, forming initial concepts.

This study eventually generated a total of 892 initial concepts, covering a wide range of opinions from university educators on collaborative climate, innovative work behaviors, and knowledge sharing. These initial concepts incorporate but are not limited to “positive collaborative climate,” “interdisciplinary cooperation,” “academic exchange and knowledge sharing,” “management support,” “innovative research method,” “resource constraints,” “knowledge exchange between colleagues,” “knowledge sharing promoting team innovation” etc. Each of these concepts reveals the unique understanding and perspective of different educators on specific topics.

#### 4.5.2 Core concepts

When categorizing the initial concepts, their logical connections were considered. For example, if multiple initial concepts pertained to interdisciplinary cooperation, these concepts were categorized under “Interdisciplinary Research Cooperation.” In setting the main categories and subcategories, this study ensured that these classifications aligned with the overall goals and research questions. For example, the main categories of “Collaborative Climate” and “Knowledge Sharing” directly relate to the study’s core themes of collaboration and innovation in universities. Through these steps, this study refined a large amount of initial concepts into a more structured and organized set of information, forming a core coding table. This not only provided a deeper understanding of university educators’ viewpoints but also revealed the key factors driving collaboration, innovation, and knowledge sharing among university educators (Tables 6–8).

**Table 6:** Analysis of direct and indirect effects.

Paths	Standardized coefficient	Lower	Upper	*p*
Direct effect Educator collaborative climate → innovative work behavior	0.420	0.148	0.620	0.011
Indirect effect Educator collaborative climate → knowledge sharing → innovative work behavior	0.286	0.123	0.555	0.002
Total effect Educator collaborative climate → innovative work behavior	0.705	0.628	0.773	0.000

**Table 7:** Core coding table.

Categories	Main category	Category	Category meaning
Collaborative climate	Positive Collaboration Culture	Colleague and Management Support, Academic Freedom and Resources	Provision of internal support and resources within the university, creating a positive collaborative climate.
	Interdisciplinary Research Cooperation	Interdisciplinary Collaboration, Collaborative Project Examples	Facilitating cooperation between different disciplines, providing opportunities for innovative research.
Innovative work behavior	Innovative Research Methods	Technological Learning Challenges, Innovation Incentives	Adoption of new technologies and methods for research, encouraging innovative endeavors.
	Innovative Teaching Methods	Curriculum Design Challenge, Teaching Cooperation	Implementation of new teaching methods, integration of course content and structure.
Knowledge sharing	Participation in Academic Activities	Knowledge Sharing, Professional Development Opportunities	Sharing knowledge through academic conferences and seminars, offering opportunities for professional growth.
	Academic Learning	Informal Exchanges, Interdisciplinary Interactions	Absorbing new knowledge through reading and informal discussions, fostering understanding and integration across disciplines.

**Table 8:** Selective coding table.

Category	Main category	Associated categories
Collaborative climate	Positive Collaboration Culture	Innovative Research Methods, Innovative Teaching Methods
	Interdisciplinary Research Cooperation	Participation in Academic Activities, Academic Learning
Innovative work behavior	Innovative Research Methods	Positive Collaboration Culture, Interdisciplinary Research Cooperation
	Innovative Teaching Methods	Participation in Academic Activities, Interdisciplinary Communication
Knowledge sharing	Participation in Academic Activities	Innovative Research Methods, Innovative Teaching Methods
	Academic Learning	Positive Collaboration Culture, Interdisciplinary Research Cooperation

#### 4.5.3 Concept selection

A positive collaboration culture is closely connected to innovative research concepts. New research methods often originate from interdisciplinary cooperation and a positive collaboration culture, supporting Hypothesis H1 (The collaborative climate among university educators in Hebei province has a significant positive impact on innovative work behavior). Interdisciplinary cooperation provides a platform for knowledge sharing, supporting Hypothesis H2 (The collaborative climate among educators has a significant positive impact on knowledge sharing). New teaching methods often arise from knowledge sharing and interdisciplinary interactions. The sharing of knowledge through participation in academic activities fosters the development of innovative research and teaching methods, supporting Hypothesis H3 (Knowledge sharing has a significant positive impact on innovative work behavior). Academic learning is enhanced through a positive collaborative climate and interdisciplinary projects, supporting Hypothesis H4 (Knowledge sharing significantly mediates the relationship between the collaborative climate among educators and innovative work behavior).

## 5 Research discussion

### 5.1 Quantitative research discussion

The research results indicate that collaborative climate has a significant positive impact on innovative work behavior. Therefore, hypothesis H1 is supported. The results are consistent with the findings of Holtzman (2014), Hogan and Coote (2014), and Kumar et al. (2016). Educators generally perceive a positive collaborative climate within their universities. This includes support among colleagues, resources given by management, and encouragement of academic freedom, which significantly promote the development of innovative research methods and teaching innovations. This suggests that a positive collaborative climate not only encourages communication and collaboration among educators but also inspires them to adopt and develop new methods and technologies in research and teaching practices (Tan, 2016; Vangrieken et al., 2015).

The research results indicate that the collaborative climate among educators has a significant positive impact on knowledge sharing. Therefore, hypothesis H2 is supported. These findings are consistent with Sveiby and Simons (2002), Kumar et al. (2016), and Lei et al. (2019). The university’s collaborative climate not only provides opportunities for knowledge sharing but also fosters professional development and interdisciplinary communications (Lin and Lee, 2017). Additionally, informal discussions and exchanges play a crucial role in daily operations. Interdisciplinary collaboration projects have become a significant platform for knowledge sharing, providing teachers with opportunities to exchange ideas and collaborate (Santaolalla et al., 2020).

The research results indicate that educators’ knowledge sharing has a significant positive impact on innovative work behavior. Therefore, hypothesis H3 is supported. These findings are consistent with DeCusatis (2008), Barczak et al. (2010), and Peters et al. (2010). Investigating the impact of knowledge sharing among university teachers in Hebei province on innovative work behavior, this study finds that educators acquire new knowledge and inspiration through participating in academic activities and reading academic journals, which directly fosters their innovative endeavors in research and teaching. For instance, through interdisciplinary discussions and collaborations, educators can adopt new ideas and technologies in teaching methods and research practices. Therefore, it can be argued that knowledge sharing plays a critical role in promoting innovative work behaviors (Wang et al., 2017).

Examining the mediating effect of knowledge sharing among university educators in Hebei province on the relationship between the collaborative climate and innovative work behaviors, it was found that knowledge sharing acts as a bridge between the collaborative climate and innovative work behaviors among university educators. Therefore, hypothesis H4 is supported. These results are similar to the findings of Kucharska and Kowalczyk (2016). In a positive collaborative climate, educators are more willing to share and absorb new knowledge, and this sharing further promotes innovation in teaching and research (Kucharska and Kowalczyk, 2016). For example, by participating in interdisciplinary seminars and collaborative projects, teachers not only enhance their mutual understanding but also jointly explore new research directions and teaching methods. This indicates that knowledge sharing plays a key mediating role between the collaborative climate and innovative work behaviors among university educators.

### 5.2 Qualitative research discussion

Under a positive collaborative climate, interdisciplinary research cooperation and participation in academic activities have promoted innovative research methods and teaching innovations. Such climate not only facilitates academic learning but also encourages exchanges across disciplines, thereby further strengthening the implementation of knowledge sharing. Through innovative work behavior, such as innovative research methods and teaching innovations, the role of knowledge sharing is enhanced in participation in academic activities as well as academic learning. These interconnected categories reflect a close relationship between collaboration, innovation and knowledge sharing in the academic environment, jointly advancing academic research and education.

## 6 Research conclusions

This study explores the impact of collaborative climate on educators’ innovative work behavior in higher education institutions. The results show that educators generally perceive a positive collaborative climate within the universities, which includes peer support, resource provision from management, and encouragement of academic freedom. This significantly promotes the development of innovative research methods and the innovation of teaching approaches. It indicates that a positive collaborative climate not only encourages communication and collaboration among educators but also inspires them to adopt and develop new methods and techniques in research and teaching practices (Tan, 2016; Vangrieken et al., 2015).

This study also investigates the impact of collaborative climate on knowledge sharing among educators in higher education institutions. The collaborative climate within the schools not only provides opportunities for knowledge sharing but also promotes professional development and interdisciplinary communication (Lin and Lee, 2017). Additionally, informal discussions and exchanges play an important role in daily work. Interdisciplinary collaborative projects have become key platforms for knowledge sharing, providing educators with opportunities to exchange ideas and collaborate (Santaolalla et al., 2020).

The study examines the impact of knowledge sharing on educators’ innovative work behavior and finds that educators acquire new knowledge and inspiration by participating in academic activities and reading academic journals, which directly promotes their innovative attempts in research and teaching. For example, through interdisciplinary discussions and collaborations, educators are able to adopt new ideas and techniques in their teaching methods and research practices. Therefore, knowledge sharing is considered to play a crucial role in fostering innovative work behavior (Wang et al., 2017).

The study explores the mediating role of knowledge sharing between collaborative climate and educators’ innovative work behavior, revealing that knowledge sharing acts as a bridge between the collaborative climate and innovative work behavior of educators in higher education institutions. In a positive collaborative climate, educators are more willing to share and absorb new knowledge, and this knowledge sharing further promotes innovation in teaching and research (Kucharska and Kowalczyk, 2016). For instance, by attending interdisciplinary seminars and collaborative projects, educators not only enhance mutual understanding but also jointly explore new research directions and teaching methods. This demonstrates that knowledge sharing plays a key mediating role between collaborative climate and educators’ innovative work behavior.

## 7 Research suggestions

### 7.1 Optimizing educator collaborative climate in multi-dimensions

For universities, the capability of establishing a shared vision, encouraging members to participate in the discussion and formulation of development policies is crucial, thereby enhancing educators’ sense of belonging and spirits of cooperation. For the administrations, the first step should be establishing a positive collaborative culture, clarifying the importance of cooperation. Through public promotions and training, educators can fully recognize the crucial role of collaboration in teaching, research, and personal growth, fostering a common value orientation. Additionally, creating a conducive collaborative environment and providing resource support are essential. Offering necessary resources such as available places, equipment, and fundings for cooperation can reduce the costs of collaboration and enhance efficiency. Establishing incentive mechanisms to reward educators who achieve significant outcomes in collaborations can also motivate them for further engagement and enthusiasm for collaborative effects.

### 7.2 Universities promoting knowledge sharing through resources and platforms

From universities’ perspectives, firstly, they should establish a cultural climate for knowledge sharing, where levels of educators’ knowledge sharing can be guided and promoted through university lectures, seminars, and other forms of engagement. Universities should strive to create an environment that encourages knowledge sharing, including intensifying awareness efforts to make all faculty deeply understand the importance of knowledge sharing and establish a consciousness about it. Strengthening the promotion of the importance of knowledge sharing, guiding educators to embrace the concept, and recognizing its crucial relevance for personal growth, university development, and academic progress. Moreover, the establishment of organizational culture for mutual trust, encouragement of healthy competition are also important for improving the effectiveness of knowledge sharing.

### 7.3 Universities providing hardware and software resources for optimizing educator innovative work behavior

In terms of hardware resources, it is essential to guarantee the timely advancement of teaching equipment and laboratory facilities. This includes the introduction of industry-leading instruments the Hebei province, ensuring the educators can conduct experimental and practical teaching activities conveniently. Focuses should also be shared to improve classroom environments by upgrading projectors, sound systems, and incorporating interactive whiteboards and other technological teaching tools to make teaching more engaging and efficient. Regarding software resources, the focus should be on improving teaching management systems and online learning platforms. These platforms can help the lecturers better manage courses, assign homework, and interact and communicate with students online. Additionally, providing access to research management software and databases will greatly improve research efficiency, aiding researchers more effectively in literature reviews, data analysis, and thesis writing.

## 8 Research limitations and future prospects

The first limitation is the scope of the study, which was based solely on a sample of educators from four universities. This may not sufficiently over all relevant groups of educators, thus affecting the stability and generalizability of the research results. In order to gain a more comprehensive understanding of the innovative work behaviors of university educators in North China, it is necessary to expand the size of sampling to include more types of institutions and a more diverse group of members in future research, in order to enhance its representativeness and accuracy.

A second limitation is the neglect of other potential contributing factors such as personal characteristics of educators, innovation capacity and open minds to new ideas etc. These are all key factors that shape their innovative work behavior, while these traits determine whether educators can think about and solve problems from new perspectives and approaches, thus affecting their actual innovative practices. To enhance the comprehensiveness and accuracy of the research, future studies should explore potential influencing factors such as teachers’ personal traits, school organizational culture, and policy environment in greater depth. First, teachers’ personal traits are a variable that cannot be overlooked (Mubarak et al., 2021), which includes their personality characteristics, cognitive styles, motivations, and values. Additionally, psychological factors such as teachers’ self-efficacy, job satisfaction, and perceptions of innovation may also significantly impact their Innovative Work Behavior (Wei et al., 2020). Furthermore, the policy environment of schools, including teacher evaluation systems, incentive mechanisms, and resource allocation, can also affect teachers’ Innovative Work Behavior (Peng et al., 2021).

## Data Availability

The original contributions presented in the study are included in the article/supplementary material, further inquiries can be directed to the corresponding author.
